# Evaluation of ChatGPT’s Accuracy, Repeatability, and Reasoning Ability in Prosthodontics Education: A Cross-Sectional Comparative Study with Prosthodontists

**DOI:** 10.4317/jced.63583

**Published:** 2026-02-26

**Authors:** Naila Perween, Punit Raj Singh Khurana, Anju Aggarwal, Aditya Chaudhary, Kartika Nitin Kumar, Sahba Hassan, Athulya R. Sekhar

**Affiliations:** 1 Associate Professor, Department of Prosthodontics, ITS Dental College, Hospital and Research Centre, Greater Noida, Uttar Pradesh- 201310; 2 Professor and Head, Department of Prosthodontics, ITS Dental College, Hospital and Research Centre, Greater Noida, Uttar Pradesh- 201310; 3 Professor, Department of Prosthodontics, ITS Dental College, Hospital and Research Centre, Greater Noida, Uttar Pradesh- 201310; 4 Assistant Professor, Department of Prosthodontics, ITS Dental College, Hospital and Research Centre, Greater Noida, Uttar Pradesh- 201310; 5 Tutor, Department of Prosthodontics, ESIC Dental College &amp; Hospital, New Delhi- 110085

## Abstract

**Background:**

The integration of artificial intelligence (AI) tools like ChatGPT in dental education is increasing, yet their accuracy, reasoning quality, and reliability remain underexplored in specialized fields like prosthodontics. This study aimed to evaluate the performance of ChatGPT in answering prosthodontics-based questions by comparing its accuracy with that of experienced Prosthodontists, as well as assessing its repeatability and reasoning ability.

**Material and Methods:**

A cross-sectional observational study was conducted using 36 validated prosthodontics-based questions, categorized by difficulty (easy, medium, hard) and type (theoretical, clinical). Responses were obtained from a panel of Prosthodontists via Google Form and from ChatGPT 4-o mini version, twice daily for 15 days. Each group generated 1080 responses. Accuracy of ChatGPT's responses was compared with Prosthodontists' responses. ChatGPT's reliability was assessed using Intraclass Correlation Coefficient (ICC), Standard Error of Measurement (SEM), and Coefficient of Variation (CV). Five subject matter experts rated ChatGPT's reasoning quality on a 3-point Likert scale, and Pearson correlation was used to analyze the relationship between reasoning and accuracy.

**Results:**

Prosthodontists outperformed ChatGPT in overall accuracy (p &lt; 0.05), with significant differences observed particularly for medium-difficulty and clinical questions. ChatGPT demonstrated fair reliability (ICC = 0.427), with SEM of 25.18 and CV of 61.7% indicating moderate variability. Reasoning analysis showed that 38.9% of ChatGPT's responses were rated strong, while 36.1% were rated poor. A significant positive correlation was found between reasoning quality and accuracy (r = 0.353, p = 0.035).

**Conclusions:**

ChatGPT demonstrates moderate ability in delivering accurate theoretical information but lacks consistency and clinical judgment. Its role should be limited to a supplementary aid in dental education, with expert oversight required to ensure accuracy and contextual relevance.

## Introduction

Within the realm of healthcare, the advent of Artificial Intelligence (AI)-powered technologies has opened new avenues for enhancing diagnosis, treatment planning, and overall patient care (1). Large language models (LLMs) represent a paradigm shift in AI, particularly in natural language processing. Large Language Models (LLMs) are advanced artificial intelligence systems trained on vast amounts of text data, allowing them to grasp the nuances, context, and complexities of language. The integration of artificial intelligence (AI) into various fields has transformed the landscape of research, education, and practice (2). Among these advancements, the emergence of conversational Generative AI models, such as ChatGPT, presents a unique opportunity to explore the intersection of technology and dentistry. From diagnosis of dental conditions, disease prediction, prognostic evaluation, and clinical decision-making, AI has seen multiple applications in dentistry (3). Chat Generative Pre-Training Transformer or ChatGPT is currently one of the most popular natural language processing (NLP). Developed by Open AI (OpenAI, L.L.C., San Francisco, CA, USA), and launched in November 2022, Chat GPT showcases promising potential to reshape the landscape of dentistry. This powerful tool excels in understanding and generating human like text using deep learning and neural networks, making it a versatile companion for a wide range of tasks (2). ChatGPT-4o mini is free and easily accessible by the public, thus making it a great success. The performance of AI has been evaluated in various healthcare disciplines, such as Rheumatology (4), Ophthalmology (5), Hepatology (6), etc. However, in the field of dentistry, the existing literature is sparse, and this necessitates the need for ChatGPT to be studied comprehensively. A systematic review highlighted the promising potential of AI models in implant type recognition, success prediction, and design optimization (7). Despite this, there remains a gap in evaluating ChatGPT's performance in providing specific answers pertinent to the specialty of prosthodontics. Previous studies on ChatGPT have assessed the accuracy and repeatability of ChatGPT in few fields of dentistry but its clinical application-based knowledge remains to be studied (8 - 9). Hence, it's imperative to meticulously evaluate ChatGPT's performance within the domain of prosthodontics to ascertain its reliability and reproducibility, thereby encouraging a discerning approach towards its potential utilization. This study endeavors to contribute to existing literature at the intersection of artificial intelligence and prosthodontics. By fostering dialogue and critical inquiry, we aim to facilitate informed decision-making among dental professionals, researchers, and stakeholders, ultimately advancing the integration of AI into the evolving landscape of modern dentistry. The research hypothesis (H1) was that ChatGPT will be accurate in answering both clinical and theoretical application-based questions. Whereas, Null hypothesis (H0) stated that ChatGPT will not be accurate in answering both clinical and theoretical application-based questions.

## Material and Methods

Question Dataset: A pool of 48 binary questions was initially developed following guidelines published by the British Society for the Study of Prosthetic Dentistry (10). The questionnaire encompassed all domains of Prosthodontics with both clinical and theoretical based questions. After an initial pilot testing and refinement for grammar and clarity, 36 questions were finalized. These were validated by four internal and 2 external subject matter experts (SME) for content and face validity. Individual item (question) was scored by SME for relevance (Scale 1-4), clarity (Scale 1-4) and completeness (Scale 1-4) and face validity (Scale 1-5). I-CVI, S-CVI/Ave, S-CVI/PR, and S-CVI/UA all exceeded 0.83, and impact scores were &gt;1.5, confirming acceptable validity. Based on these calculations, it was concluded that the scale of questionnaire has achieved satisfactory level of content validity and face validity. The answers for each question were obtained from standard textbooks and reference articles which were further validated through discussion between six experts working as faculty in the concerned specialty. Categorization of Questions: All 36 questions were categorized into theoretical and clinical application-based questions (18 questions each). The questions are also classified into three levels of difficulty: Easy, Medium, and Hard (12 questions each). Two prosthodontists were involved in the categorization of the questions. When difference of opinion arose in certain questions, they were resolved by a third prosthodontist. Generation of Answers in ChatGPT: Questions were entered into the web version of ChatGPT-4o mini by a single general dentist to ensure consistency and minimize bias. Each question was asked twice daily (morning and evening) by opening "new chat" for a period of 15 days. All questions were followed with the prompt "Only Yes or No as answer" or "Only True or False as answer" or "Choose only one as answer". All answers generated by ChatGPT were entered into an Excel spreadsheet. The same investigator further prompted ChatGPT with "How" or "Why" for reasoning, which was evaluated by five experts on 3-Point Likert Scale (0-Incorrect, 1-Parial/Incomplete, 2- Correct). Acquisition of Answers from Prosthodontists: To acquire responses from Prosthodontists, all questions were entered into a Google Form and circulated among 30 Prosthodontists, working as faculty in the concerned department. The answers generated by them were recorded in an Excel Spreadsheet. Statistical Analysis: Data entered into Microsoft Excel spreadsheet was coded and subjected to statistical analysis using IBM SPSS version 21.0 software. The responses to the questionnaire were dichotomized for statistical analysis and better interpretability. Shapiro Wilk test was used to check which all variables were following normal distribution. Data was normally distributed; therefore, inferential statistics were performed using parametric test. For Intergroup comparison, Independent t test was used. Accuracy was assessed using Wald- binomial method, while reliability was assessed using Intraclass Correlation Coefficient. Reasoning ability graded on 3- point Likert Scale was analyzed and further subjected to Pearson's Correlation Analysis. The result is considered significant at p&lt;0.05 at 95% confidence interval (CI).

## Results

Thirty Prosthodontists and ChatGPT generated 1080 answers each for a set of 36 questions. A statistically significant difference was observed in the overall accuracy between Prosthodontists and ChatGPT (p&lt;0.05), with Prosthodontists demonstrating higher accuracy, (Table 1).

Table 1

**Figure 1:**
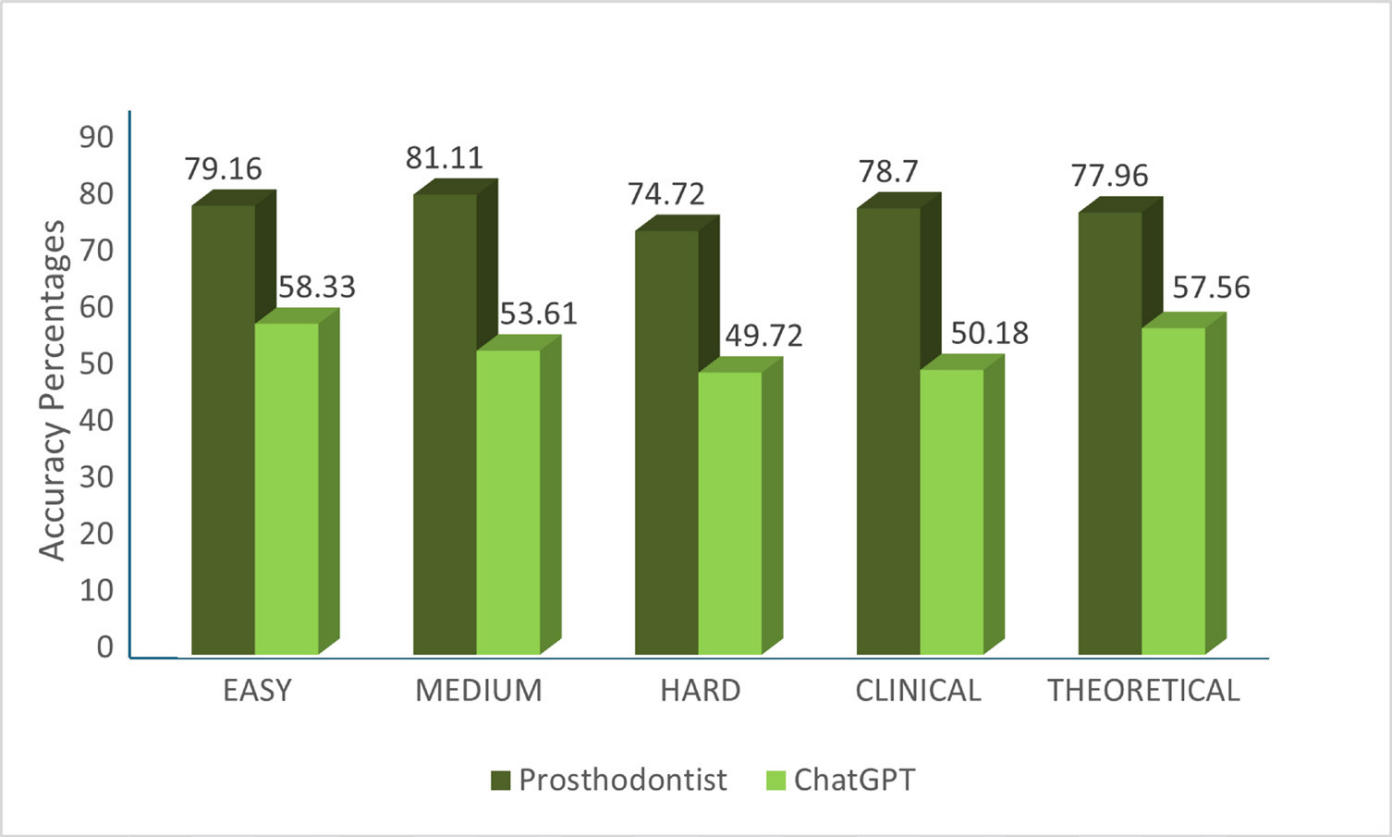
Distribution of accuracy scores of ChatGPT in comparison with Prosthodontists across varying question difficulties (easy, moderate, difficult) and types (theoretical vs. clinical).

When accuracy was compared based on question categories: For easy and hard questions, Prosthodontists had higher mean accuracy scores than ChatGPT; however, the differences were not statistically significant (p&gt;0.05). Statistically significant differences were observed in the accuracy between Prosthodontists and ChatGPT as p&lt;0.05 when assessed for medium questions. Prosthodontists demonstrated statistically significant higher accuracy in clinical questions. Higher accuracy was observed for theoretical questions by Prosthodontists, but the difference was not statistically significant as p&gt;0.05, (Fig. 1).

1

The test-retest reliability of ChatGPT's responses was evaluated using Intraclass Correlation Coefficient (ICC3,1), (Table 2).

Table 2

**Figure 2:**
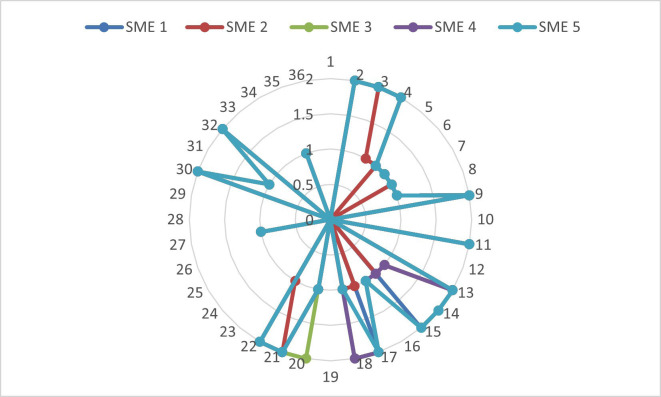
Variability of Likert Scale grades of Subject Matter Experts across all 36 questions.

The ICC value was 0.427 (95% Confidence Interval ranging 0.321 to 0.565), indicating fair reliability according to Cicchetti's guidelines (11). The Standard Error of Measurement (SEM) was approximately 25.18, which implies a moderate degree of measurement error in the repeated responses by ChatGPT. The Coefficient of Variation (CV) was 61.7%, reflecting substantial relative variability in the accuracy scores. These results suggest that ChatGPT's performance on the 36-item questionnaire is moderately consistent, but not highly reliable according to previous studies (12 - 13). Differential statistics was performed on the Likert Scale scores given by 5 Subject Matter Experts on the reasoning ability of ChatGPT, (Fig. 2).

2

**Figure 3:**
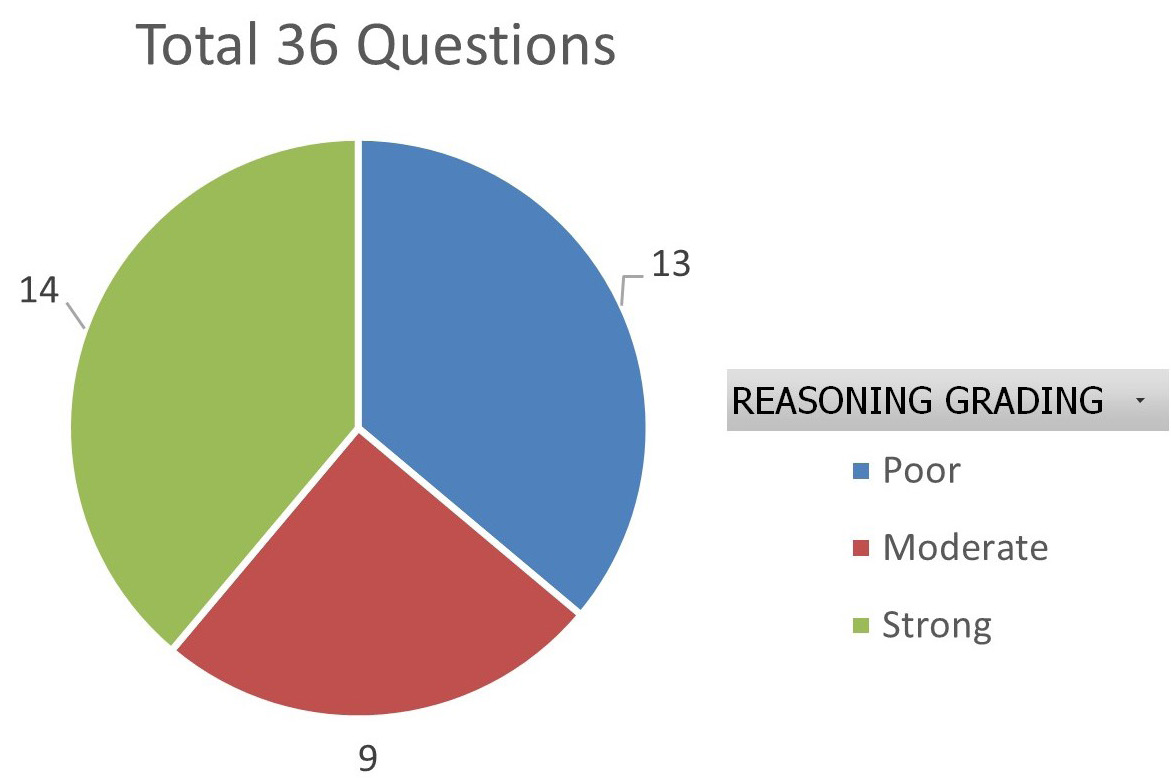
Reasoning Ability distribution across all 36 questions.

Out of 36 total questions evaluated, responses to 14 questions (38.9%) were rated as demonstrating strong reasoning, with a mean score 1.5, and minimal inter-rater variability (SD = 0-0.55). These responses typically displayed clear, contextually appropriate, and logical justifications. In contrast, responses to 9 questions (25%) were assigned moderate reasoning scores. These responses reflected partially logical explanations and were sometimes ambiguous or inconsistent in reasoning. These had mixed SME ratings (primarily 1s with occasional 0s or 2s), and moderate SD values. Furthermore, 13 questions had responses (36%) which received a poor reasoning rating (mean score &lt; 0.5), with unanimous SME agreement in several cases. These answers lacked relevance, coherence, or displayed factual inaccuracies, (Fig. 3).

3

To further analyze the relationship between ChatGPT's reasoning quality and its response accuracy, Pearson's correlation analysis was conducted. The results revealed a statistically significant positive correlation, with Pearson's r = 0.353 and a p-value of 0.035 (95% Confidence Interval: 0.03 to 0.61), (Table 3).

Table 3

This indicates that as the reasoning quality of ChatGPT's responses improved the likelihood of the answers being accurate also increased. Although the correlation strength was low to moderate, it was nonetheless statistically meaningful.

## Discussion

The findings of the present study provide a multi-faceted analysis of ChatGPT's proficiency in the field of Prosthodontics. Given ChatGPT's ability to generate nuanced responses based on vast data, this study aimed to assess its accuracy, repeatability and reasoning in answering both clinical and theoretical questions in prosthodontics. The goal was to evaluate whether such technology could serve as a reliable, supplementary resource in dental education and practice. The research hypothesis that the answers generated by ChatGPT would be more accurate or repeatable was rejected, as ChatGPT-4o mini demonstrated limited ability in both accuracy and repeatability. ChatGPT-4o mini performed with an average of 53.88% accuracy compared to 78% by prosthodontists. This is assumed to be because of the probabilistic nature of LLMs which results in variable answer generation (14), hence for each question, 30 answers were obtained from ChatGPT. This also helped the authors assess the repeatability which was found to be 51%. The results of this study aligns with the findings from a comparative study between ChatGPT and endodontic experts, which found ChatGPT to be 57.33% accurate, however ChatGPT was found to be 85.4% consistent for similar dichotomous questions, which is higher from that observed in the present study (15). Another study which evaluated performance of ChatGPT in answering removable prosthodontics and fixed prosthodontics questions reported only 25.6% accuracy (14). The statistically significant difference in overall accuracy in favor of Prosthodontists confirms that human experts continue to outperform AI models in domain-specific tasks that require specialized training and experiential knowledge. It was observed that AI models perform best with structured, knowledge-based tasks rather than context-specific, clinically nuanced scenarios (16). In this study, ChatGPT's performance was poorer on clinical questions than theoretical ones, and this difference was statistically significant. This highlights a potential limitation in AI's contextual understanding of clinical scenarios. This supports previous research from medicine indicating that while AI can process well-defined information, it struggles with clinical complexities that require human judgement and contextual reasoning (17). A diagnostic accuracy threshold &gt;90% accuracy has been recommended for clinical reliability in artificial intelligence systems (18). Such performance has been observed in licensing exam-style questions, where ChatGPT performs well (16 , 19 - 20). ChatGPT has shown strong performance in structured, image-based diagnostic tasks. Mago et al. reported 100% accuracy for selected oral and maxillofacial radiology queries (21), while Ahmed et al. observed diagnostic accuracies exceeding 90% for AI-based dental caries detection (22). In contrast, a systematic review by Mohammad-Rahimi et al. reported a wider accuracy range of 71%-96% across AI-driven dental diagnostics, reflecting variability related to task complexity and data type (23). However, its clinical application is inconsistent. A study on endo-oral lesion detection reported limited clinical usability, indicating that although artificial intelligence performs well in image interpretation, a structured domain, it may underperform in scenarios requiring integrative clinical judgment (24). This discrepancy highlights that while AI excels in visually guided tasks, its application in clinical prosthodontic decision-making remains limited, as such scenarios demand a synthesis of hands-on expertise and nuanced judgment that ChatGPT may not yet reliably replicate. Reliability analysis revealed ChatGPT's limited consistency, with an ICC value of 0.427 which denotes "fair" reliability as per Cicchetti's guidelines (11). The relatively high SEM and CV values further illustrate substantial variability across responses, highlighting the unpredictability of LLM output. This stochastic nature of output generation, driven by its underlying probabilistic model, raises concerns about the reliability of AI-generated responses, particularly in professional and educational settings where consistency is paramount (19). The SME evaluation of reasoning quality added another layer of insight into ChatGPT's functional capabilities. Less than 40% of answers demonstrated strong reasoning, while over one-third showed poor reasoning. This underscores deficiencies in coherence, especially in context-driven clinical queries. A modest but significant correlation (r = 0.353) between reasoning and correctness suggests that logical justifications correlate with higher accuracy. This finding emphasizes the importance of evaluating the explanatory quality of AI responses rather than focusing solely on the correctness of the final answer. Notably, ChatGPT was able to generate completely correct or partially correct reasoning even for those questions in which ChatGPT's repeatability was very low for the correct answer. For example, for the question "During border moulding, is swallowing advocated for recording the distolingual border of mandible?", the correct answer was given only 5 out of 30 times (16.67% repeatability), yet correct reasoning was given when specifically prompted. Similarly, for the "Shaped Blockout" question, ChatGPT always answered incorrectly but gave correct reasoning when asked "How". These examples reveal that ChatGPT's reasoning ability may be prompt-dependent, relying heavily on the input's phrasing and structure (25). ChatGPT struggled when specific clinical keywords were missing. For example, in a clinical question "Patient has missing 36 and 37. Can we give fixed bridge connecting natural teeth 35 as abutment and implant supported abutment on 37, without using stress breaker?"; ChatGPT was unable to answer correctly when stress breaker was not mentioned. These observations support prior research that highlights ChatGPT's dependence on clear, specific prompts (16 , 26). Dental students, who are frequent users, may fail to craft optimal prompts, leading to misinformation or confusion. ChatGPT also underperformed with questions involving numerical values. For example, in a question "Metal ceramic restoration requires functional reduction of a. 1.5-2mm/ b. 1-1.5mm", ChatGPT gave contradictory answers with identical reasoning for both correct and incorrect answer. Another concern was observed with dichotomous theoretical questions. For instance, ChatGPT provided opposite explanations for an easy question- "Attachment between porcelain tooth and denture base resin is a. Mechanical/ b. Mechanochemical". Such inconsistency indicates a lack of true conceptual and contextual understanding.(19) When challenged with prompts like "Are you sure?", ChatGPT sometimes changed correct answers, as observed in the question "Massetric notch is recorded because of a. action of masseter on buccinators/ b. action of buccinator on masseter". This reflects its tendency to adjust based on perceived user feedback, not on verifiable facts. This behavior underscores the importance of understanding ChatGPT's limitations in knowledge retrieval and conversational dynamics. ChatGPT often generated detailed yet incorrect responses for clinical questions, showing that it can simulate expertise without genuine accuracy. ChatGPT can generate plausible-sounding explanations because it mimics the tone and structure of expert knowledge, even when the content is incorrect. The model cannot cross-check its answers with authoritative sources, so it sometimes "hallucinates" details that sound credible but lack basis. Since ChatGPT doesn't access authoritative sources in real time, its responses depend on pre-learned data, which may be incomplete or outdated (27). As an extension of the study, ChatGPT was re-evaluated for reasoning ability after three months. It provided improved responses in two instances, possibly due to periodic model updates and refinements (28). Kochanek et al., 2024 specifically highlighted that while newer versions show improved performance, the "great variability of responses casts doubt on possible professional applications.". This suggests that changes in ChatGPT's interface and usage limits over time may indeed introduce inconsistencies that could impact user experience and reliability (29). For example, a question on a 2017 classification of adjacent implants was answered correctly by an older version, but not by the updated one, likely because that data was not retained or accessed in real time. Although the newer versions claim browsing and external search capabilities, limitations remain. If data isn't online or if the integrated search isn't activated, the model reverts to pre-trained knowledge (28). These restrictions, including version fallback and rate limits, introduce inconsistency and complicate reproducibility in academic or clinical settings. Nonetheless, the observed improvement in reasoning suggests that periodic updates are making ChatGPT more robust. While it does not learn continuously like humans, version-level refinements enable incremental enhancements, offering promise for its future role in complex domains. It is advised that ChatGPT be utilized under the supervision of an expert who can discriminate between accurate and false material. Its tendency to present inaccurate information confidently can mislead non-expert users. Especially in prosthodontics, where errors can compromise patient outcomes or learning, careful validation is essential. Limitations of the study include the use of binary questions which may have oversimplified complexity of clinical decision making. The sample size of 36 questions, though diverse in content and structure, restricts generalization. Furthermore, ChatGPT was not tested for real-world clinical environments where factors like patient history and tactile feedback influence decision-making. Also, only one version of ChatGPT was tested, and over a limited 15-day period. Given the rapidly evolving nature of LLMs, results may vary significantly with future versions or interfaces. Future studies should expand the question pool, include real-time patient scenarios, and explore newer GPT versions. Incorporating multidisciplinary prosthodontic scenarios and updates aligned with contemporary literature may better evaluate ChatGPT's potential in clinical applications.

## Conclusions

The study suggests that ChatGPT may have utility as a supplementary tool for students and educators in prosthodontics. It can serve as an accessible platform for fact-based query resolution and content generation. However, given its inconsistent reliability, occasional lapses in reasoning, and relatively poor performance in clinical and intermediate difficulty questions, ChatGPT should be used cautiously and always under expert supervision.

## Figures and Tables

**Table 1:** Comparison of Accuracy between Prosthodontists and ChatGPT.

		N	Mean	S.D	S.E Mean	CI 95% (Wald Bionomial)	T value	P value
OVERALL ACCURACY	Prosthodontist	36	78.33	16.97	2.83	72.78%- 83.88%	3.29	0.001
	ChatGPT	36	53.89	33.2	5.54	43.03%- 64.75%		
EASY QUESTIONS	Prosthodontist	12	79.16	17.645	5.09	75.61%- 81.07%	1.75	0.093
	ChatGPT	12	58.3333	37.07	10.70	49.92%- 55.64%		
MEDIUM QUESTIONS	Prosthodontist	12	81.11	13.9503	4.03	79.89%- 85.09%	2.42	0.024
	ChatGPT	12	53.61	36.7755	10.61	58.71%- 63.93%		
HARD QUESTIONS	Prosthodontist	12	74.72	19.67	5.68	84.62%- 89.64%	2.56	0.018
	ChatGPT	12	49.72	27.43	7.92	53.21%- 58.61%		
CLINICAL QUESTIONS	Prosthodontist	18	78.7037	15.1307	3.566	71.71%- 85.69%	3.42	0.002
	ChatGPT	18	50.18	31.900	7.518	35.44%- 64.92%		
THEORETICAL QUESTIONS	Prosthodontist	18	77.962	18.53	4.37	69.40%- 86.53%	2.16	0.040
	ChatGPT	18	57.592	34.10	8.04	41.83%- 73.35%		

*P<0.05: Statistically significant; S.D: Standard Deviation; S.E: Standard Error; CI: Confidence Interval

**Table 2:** Reliability assessment for 30 answers of 30 questions generated by ChatGPT.

Test for Statistical Analysis	Value	Interpretation
Mean	53.89	Average accuracy across 30 attempts
Standard Deviation	33.26	Spread of accuracy scores
Intraclass Correlation Coefficient	0.42795% CI- 0.321 - 0.565	Fair reliability
Standard Error of Measurement (SEM)	25.18	Moderate degree of measurement error
Coefficient of Variation % (CV%)	61.7%	High relative variability
Pearson’s Correlation Correlation	0.35395% CI- 0.027 - 0.610	p- 0.035Statistically significant

ICC measured for 30 attempts to 36 questions. Benchmark Scale (Cicchetti, 1994):<0.40 - Poor Reliability0.40-0.59 - Fair Reliability0.60-0.74 - Good Reliability0.75-1.00 – Excellent Reliability

**Table 3:** Likert Scale Expert Grading for all questions.

Question No.	n0	n0 %	n1	n1 %	n2	n2 %	Mean SME score	Median	S.D	Reasoning grade
1	5	100	0	0	0	0	0	0	0	Poor
2	0	0	0	0	5	100	2	2	0	Strong
3	0	0	0	0	5	100	2	2	0	Strong
4	0	0	1	20	4	80	1.8	2	0.48	Strong
5	0	0	5	100	0	0	1	1	0	Moderate
6	1	20	4	80	0	0	0.8	1	0.48	Moderate
7	0	0	5	100	0	0	1	1	0	Moderate
8	0	0	5	100	0	0	1	1	0	Moderate
9	0	0	0	0	5	100	2	2	0	Strong
10	5	100	0	0	0	0	0	0	0	Poor
11	0	0	0	0	5	100	2	2	0	Strong
12	5	100	0	0	0	0	0	0	0	Poor
13	0	0	0	0	5	100	2	2	0	Strong
14	0	0	2	40	3	60	1.6	2	0.55	Strong
15	0	0	2	40	3	60	1.6	2	0.55	Strong
16	2	40	3	60	0	0	0.6	1	0.55	Moderate
17	0	0	1	20	4	80	1.8	2	0.45	Strong
18	0	0	2	40	3	60	1.6	2	0.55	Strong
19	5	100	0	0	0	0	0	0	0	Poor
20	0	0	4	80	1	20	1.2	1	0.45	Moderate
21	0	0	0	0	5	100	2	2	0	Strong
22	0	0	1	20	4	80	1.8	2	0.45	Strong
23	5	100	0	0	0	0	0	0	0	Poor
24	5	100	0	0	0	0	0	0	0	Poor
25	5	100	0	0	0	0	0	0	0	Poor
26	5	100	0	0	0	0	0	0	0	Poor
27	0	0	5	100	0	0	1	1	0	Moderate
28	5	100	0	0	0	0	0	0	0	Poor
29	5	100	0	0	0	0	0	0	0	Poor
30	0	0	0	0	5	100	2	2	0	Strong
31	2	40	3	60	0	0	0.6	1	0.55	Moderate
32	0	0	0	0	5	100	2	2	0	Strong
33	5	100	0	0	0	0	0	0	0	Poor
34	5	100	0	0	0	0	0	0	0	Poor
35	2	40	3	60	0	0	0.6	1	0.55	Moderate
36	5	100	0	0	0	0	0	0	0	Poor

*SME: Subject Mater Expert; S.D: Standard Deviation0-0.4 – Poor Reasoning0.5-1.4 – Moderate Reasoning1.5-2 – Strong Reasoning

## Data Availability

The datasets used and/or analyzed during the current study are available from the corresponding author.
